# In Vivo Study for Clinical Application of Dental Stem Cell Therapy Incorporated with Dental Titanium Implants

**DOI:** 10.3390/ma14020381

**Published:** 2021-01-14

**Authors:** Hyunmin Choi, Kyu-Hyung Park, Narae Jung, June-Sung Shim, Hong-Seok Moon, Hyung-Jun Kim, Seung-Han Oh, Yoon Young Kim, Seung-Yup Ku, Young-Bum Park

**Affiliations:** 1 BK21 Plus Project, Oral Science Research Center, Department of Prosthodontics, Yonsei University College of Dentistry, Seoul 03722, Korea; choi_forever@hotmail.com (H.C.); khyungpark@gmail.com (K.-H.P.); jnrgood1213@yuhs.ac (N.J.); jfshim@yuhs.ac (J.-S.S.); hsm5@yuhs.ac (H.-S.M.); 2 Department of Oral & Maxillofacial Surgery, Oral Science Research Institute, Yonsei University College of Dentistry, Seoul 03722, Korea; KIMOMS@yuhs.ac; 3 Department of Dental Biomaterials, Institute of Biomaterials-Implant, Wonkwang University School of Dentistry, Iksan 54538, Korea; shoh@wku.ac.kr; 4 Institute of Reproductive Medicine and Population, Medical Research Center, Soul National University, Seoul 03087, Korea; yoonykim96@gmail.com (Y.Y.K.); jyhsyk@snu.ac.kr (S.-Y.K.); 5 Department of Obstetrics and Gynecology, Seoul National University College of Medicine, Seoul 03080, Korea

**Keywords:** dental-derived human mesenchymal stem cells, titanium disc, rough surface, osteogenesis, animal study

## Abstract

The aim of this study was to investigate the behavior of dental-derived human mesenchymal stem cells (d-hMSCs) in response to differently surface-treated implants and to evaluate the effect of d-hMSCs on local osteogenesis around an implant in vivo. d-hMSCs derived from alveolar bone were established and cultured on machined, sandblasted and acid-etched (SLA)-treated titanium discs with and without osteogenic induction medium. Their morphological and osteogenic potential was assessed by scanning electron microscopy (SEM) and real-time polymerase chain reaction (RT-PCR) via mixing of 5 × 10^6^ of d-hMSCs with 1 mL of Metrigel and 20 μL of gel-cell mixture, which was dispensed into the defect followed by the placement of customized mini-implants (machined, SLA-treated implants) in New Zealand white rabbits. Following healing periods of 2 weeks and 12 weeks, the obtained samples in each group were analyzed radiographically, histomorphometrically and immunohistochemically. The quantitative change in osteogenic differentiation of d-hMSCs was identified according to the type of surface treatment. Radiographic analysis revealed that an increase in new bone formation was statistically significant in the d-hMSCs group. Histomorphometric analysis was in accordance with radiographic analysis, showing the significantly increased new bone formation in the d-hMSCs group regardless of time of sacrifice. Human nuclei A was identified near the area where d-hMSCs were implanted but the level of expression was found to be decreased as time passed. Within the limitations of the present study, in this animal model, the transplantation of d-hMSCs enhanced the new bone formation around an implant and the survival and function of the stem cells was experimentally proven up to 12 weeks post-sacrifice.

## 1. Introduction

The establishment of osseointegration is one of the key factors for the long-term success of oral implants and many previous studies have reported that osseointegration can be manipulated and improved by changing implant surface properties [1,2,3,4,5,6,7,8]. It is generally accepted that a rough surface promotes osseointegration more effectively than a machined surface by providing an increased surface area for cell attachment, resulting from an increase in the production of prostaglandin E2 and TGF-b1, which are known to promote osteoblast differentiation, leading to enhanced subsequent bone formation. [2,3] Consequently, mechanical and chemical surface treatments including sand blasting and acid etching (SLA), anodization, [4] hydroxyapatite coating, [5] plasma treatment, [6] and UV photofunctionization [7,8] have been used successfully to modify titanium implant surfaces. Recently, along with the advancement of tissue engineering techniques, many researchers have attempted to better understand the biological reaction underlying cell attachment to implant surfaces. Since human mesenchymal stem cells (hMSCs) have shown potential in the treatment of many diseases including inflammatory disease [9], diabetes [10], myocardial infarction [11] and liver cirrhosis [12], hMSCs have also been identified as a key cell type required for osseointegration and bone remodeling [13,14]. Although several investigators have evaluated the effect of implant surface treatment on osteogenic differentiation of hMSCs in vitro [15,16,17,18], there has been less extensive in vivo literature on the fate of hMSCs on differently surface treated implants for clinical application. Moreover, although it has been widely recognized that hMSCs obtained from dental origin have higher proliferation and osteogenic differentiation capacity compared with those in hMSCs obtained from the iliac crest, functional differences depending on origin of the cell source have been overlooked in many previous studies. Indeed, it can be anticipated that greater knowledge regarding the fate of hMSCs on implant surfaces, especially hMSCs obtained from dental origin, could lead to greater clinical implant success and further understanding of the underlying mechanisms governing the fate of d-hMSCs. Therefore, the aim of this study was to investigate the behavior of d-hMSCs in response to differently surface-treated implants and to evaluate the effect of d-hMSCs on local osteogenesis around an implant in vivo.

## 2. Materials and Methods

### 2.1. Titanium Surface Preparation

In brief, titanium discs with dimensions of 10 mm diameter and 2 mm thickness were fabricated using commercially pure grade IV titanium alloy (Dentium Co., Ltd., Seoul, Korea) and either machined or sandblasted with large grits and acid etched (SLA)-treated, ready to be used for the cell seeding. SLA-treated titanium discs were sandblasted with aluminum oxide powder (Al_2_O_3_)(Dentium Co., Ltd.) and acid-etched with a concentrated hydrochloric acid (HCL) 37% (Sigma-Aldrich, St. Lous, MO, USA). Prior to usage, all titanium discs were thoroughly cleaned with distilled water, sterilized and dried with ethylene oxide (EO) gas (Duksan, Seoul, Korea).

### 2.2. Cell Preparation

The experiment was approved by the Institutional Research Ethics Committee of the Yonsei University College of Dentistry (IRB NO.14-0097). Human alveolar bone marrow from mandibles was obtained from healthy subjects (aged 19–40 years old) who visited the Yonsei University Dental Hospital. To obtain d-MSCs, bone marrow from alveolar aspirates (0.5–1.0 mL) was obtained from osteotomy sites during implant surgery using an 18-gauge needle syringe without contamination of periodontal tissues. Stromal cells including erythrocytes were plated in a 100-mm tissue culture dish (BD, Pharmingen, CA, USA) and cultured in 15 mL of DMEM containing 10% FBS (Gibco, Invitrogen Corporation, Grand Island, NY, USA) and 100 units/mL penicillin G and 100 μg/mL streptomycin. During three days of cell seeding, unattached cells were withdrawn and attached cells were provided with a fresh medium. Subsequent passages were performed when cells were approximately in 80% confluence. Each cell at passages P3-P4 was used for the characterization and further evaluation. The characterization process involved immunocytochemical analysis, cell-surface-marker characterization, and induction of osteogenic differentiation to test if d-hMSCs possessed the basic characteristics of mesenchymal stem cells (data not shown).

### 2.3. Surface Analysis by SEM

The titanium surfaces were analyzed using a scanning electron microscope (SEM, Hitachi, S-800, Tokyo, Japan) at 15 Kv accelerating voltage. The samples were then photographed at 1, 5 and 7 days after initial culturing for 2 days in standard MSC culture media, followed by culturing in osteogenic-conditioned media containing 10 mM β-GP, 10 μM dexamethasone and 50 μM ascorbic acid. To obtain SEM images, the samples were then fixed in 2% glutaraldehyde-paraformaldehyde buffer, rinsed, dehydrated in graded alcohol and dried. Their time and surface-dependent morphological changes were analyzed using a fully computerized high-resolution SEM at three different magnification levels (1:12, 1:1000 and 1:3000).

### 2.4. Quantitative RT-PCR

Total RNA was isolated using an RNA RNeasy kit (Qiagen, Valencia, CA, USA). Spectrophotometry and denaturing agarose gel electrophoresis were used to determine the consistency of the quality of RNA. From 1 µg of purified total RNA, cDNA was synthesized using a PrimeScript RT reagent kit (Invitrogen), following the directions of the producer. The primer sequences used have been described in Table 1. With SYBR green real-time PCR master mix (Thermo Fisher scientific, Waltham, MA, USA), quantitative RT-PCR analysis was conducted with a 7500 Real-Time PCR System (Thermo Fisher scientific). The conditions for PCR were 95 °C for 30 s, 40 denaturation cycles for 5 s at 95 °C and annealing and extension for 15 s at 60 °C, accompanied by dissociation and a typical denature action curve.

### 2.5. Preparation of Customized Implant and Animal Preparation and In Vivo d-hMSC Transplant

Non-threaded type customized implant (Dentium Co., Ltd., Seoul, Korea) with a diameter of 1.5 mm and height of 3.7 mm were designed and surface-treated (machined or SLA treated).

**Figure 1:**
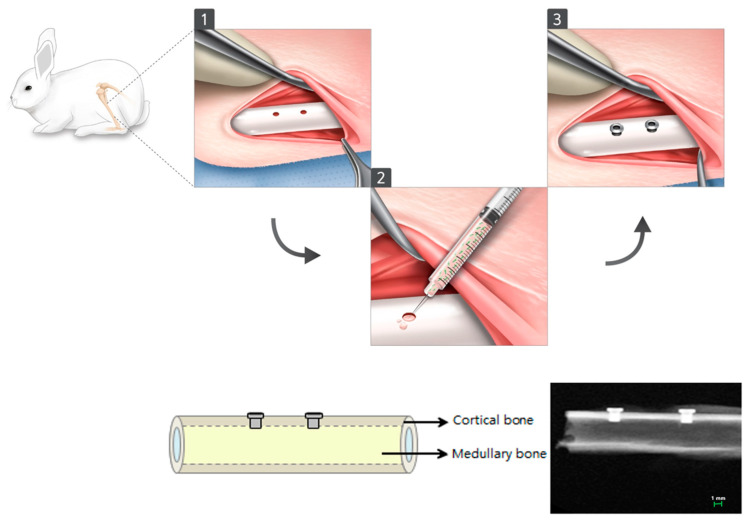
Surgical procedure used in this study.

In this study, 20 New Zealand white rabbits, 6 weeks old and weighing 3.5–4.0 kg each were used. A total of 80 samples were randomly distributed to 20 New Zealand white rabbit as 4 samples can be obtained from a single rabbit (Table 2). The Institutional Animal Care and Use Committee, Yonsei Medical Center, Seoul, Korea, reviewed and approved animal collection, management, surgical protocols and procedures for this research (Approval no. 2013-0347-2). The animals were anesthetized with a combination of 30 mg/kg of Zolazepam (Zoletil Virback Korea Co., Seoul, Korea) and 10 mg/kg of Xylazine HCI (Rumpun, Bayer Korea, Seoul, Korea) given intravenously, followed by shaving and sterilizing the surgical site with povidone-iodine. After 10 minutes, infiltration with 2% lidocaine HCl and epinephrine 1: 80,000 was given. A defect size of 1.0 mm in diameter and 4mm in height was made using a dental diamond bur and customized implants were placed in the right and left tibia of the rabbit. According to the study design, 5 × 10^6^ d-hMSCs mixed with 1ml of Metrigel^TM^ (BD) and 20 µL of gel-cell mixture were dispensed into the defect prior to implant placement. After 2 and 12 weeks post-implantation, animals were sacrificed using 2% paraformaldehyde injection to the heart under a general anesthetic condition (Figure 1). Then, for 2 weeks, block sections including implants were retained and fixed in 10% neutral buffered formalin. The samples obtained were examined radiographically, histomorphometrically and immunohistochemically, respectively.

### 2.6. Radiological Analysis

**Figure 2:**
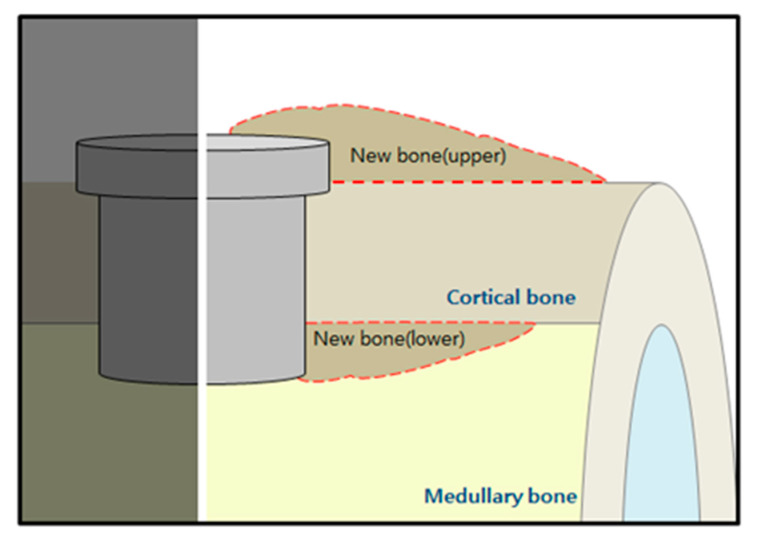
Schematic diagram depicting the measuring parameters (newly formed bone: upper and lower).

**Figure 3:**
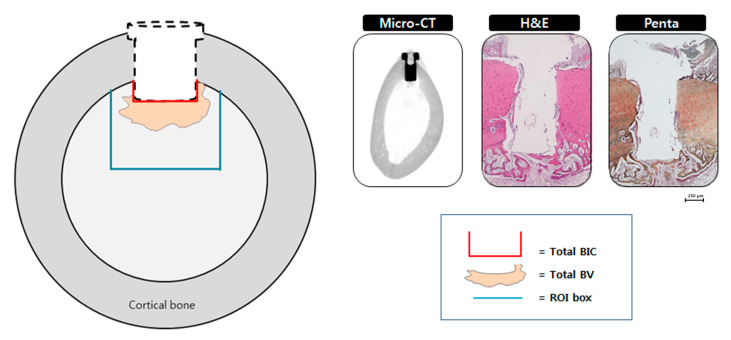
Method of calculating the bone-implant contact (BIC) value and newly developed bone area within the region of interest (ROI).

New bone volume was determined at two points from plain X-ray images: (a) new bone volume (µm × µm) formed from the implant head, (upper) and (b) new bone volume (µm × µm) formed from the apical part of the implant (lower) (Figure 2). Each measurement was performed by one experienced examiner using specific image software (Image-Pro Plus, Media Cybernetics, Silver Spring, MA, USA). 3D photographs of the samples were reconstructed and the amount of fresh bone forming around implants was determined using micro-computed tomography scanners (SkyScan 1076, Skyscan, Aartselarr, Belgium) inside the area of interest (ROI) box at a resolution of 35 um (achieved using 100 kV and 100 uA). ROI box was set with dimension of 1.30 mm (x axis) × 1.30 mm (y axis) × 0.45 mm (z axis) (Figure 3).

### 2.7. Histological and Histomorphometric Analysis (H&E, Russell-Movat Pentachrome: Bone Volume, Bone-Implant Contact) and Immunohistochemistry (Human nuclei A, BrdU)

The specimens were dehydrated for 1 week at 2 h intervals using graded alcohols of 70%, 80%, 95% and 100%. The specimens were then placed in Technovit 7200 (Heraeus Kulzer, Dormagen, Germany) and ethanol (1:3, 1:1, and 3:1 ratio) and sectioned using a diamond saw into the buccolingual plane (Exakt 300, Kulzer, Norderstedt, Germany). The central segment was reduced to a final thickness of approximately 15 μm from each implant site by micro grinding and polished with a cutting-grinding system (Exakt 400CS, Exakt Apparatebau, Norderstedt, Germany) and finally stained with hematoxylin and eosin (H&E) and pentachrome stain by Russell–Movat (American Master Tech, Lodi, CA, USA). The stained specimens were captured using a light microscope (Leica DM 2500, Leica Microsystems, Wetzlar, Germany) at ×12.5, ×50 and ×100 magnification. The new bone volume (BV) and new bone-implant contact (BIC) within the ROI box around the implants were measured by imaging analyses system (Image-Pro Plus 4.5 Media Cybernetics Inc., Silver Spring, MD, USA). The ROI box was set with dimensions of 1.30 mm (x axis) × 1.30 mm (y axis) (Figure 3).

Immunohistochemistry analysis was conducted according to a previously reported study [19]. Briefly, paraffin samples were de-paraffinized and hydrated. Then, antigen retrieval was carried out with sodium citrate buffer (10 mM Sodium citrate, pH 6.0, 0.05% Tween 20). The endogenous hyperoxidase was blocked with 0.3% H_2_O_2_ in PBS. After incubation of the sections in blocking buffer, using biotinylated anti-mouse or anti-rabbit secondary antibodies, all the bound antibodies were found, followed by the addition of complex avidin-peroxidase. Counter-staining was performed with hematoxylin. Antibody dilutions used in this study were as follows: human nuclear A antigen 1:25 (ab191181, Abcam, Cambridge, MA, USA).

### 2.8. Statistical Analysis

The results from the radiological, histomorphometrical and immunohistochemical analysis were expressed as the means +SDs and difference between groups were analyzed using SPSS (version 23.0, IBM Corporation, NY, USA). Statistical significance was analyzed by one-way ANOVA. Post-hoc Duncan’s multiple-range test was used to identify statistically significant difference, with a significance level of 5%.

## 3. Results

### 3.1. Surface Analysis by SEM

**Figure 4:**
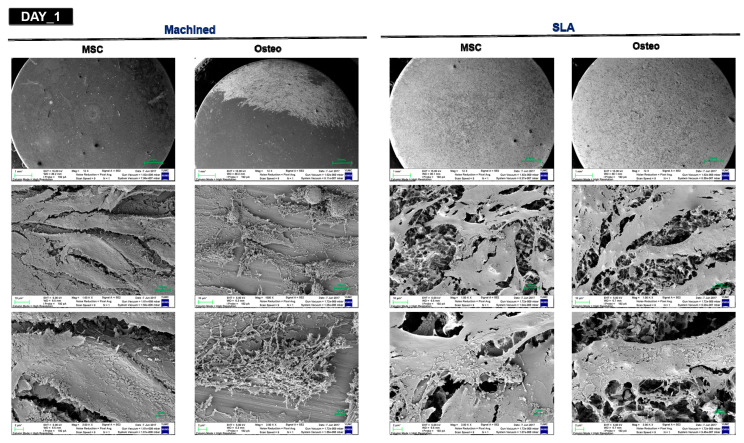

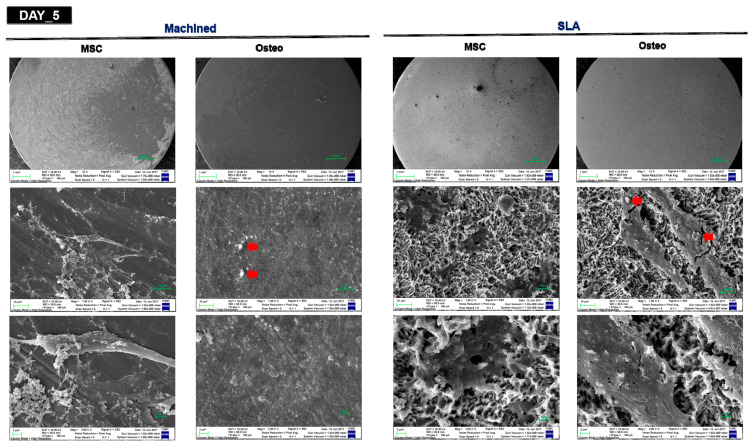

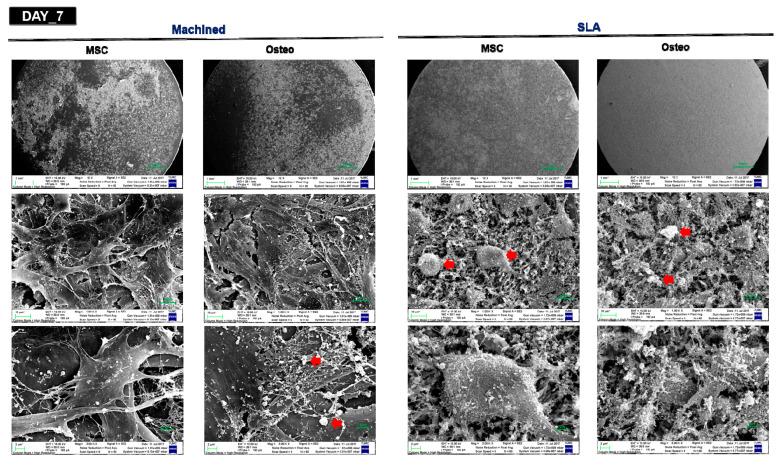
SEM images of d-hMSCs cultured with basic MSC culture medium (MSC) and osteogenic differentiation medium (osteo) on machined and SLA-treated titanium surfaces at 1, 5 and 7 days after initial culturing at three different magnification levels (upper 12×, middle 100× and bottom 3000× magnification; red arrows indicate mineralized crystalline-like structure).

The disparity in cell morphology and surface features was clearly seen from the surface of the machined and SLA-treated titanium discs. SEM analysis showed that d-hMSCs seemed to be adhered well across the whole surface from day 1 regardless of the surface type and use of osteogenic induction media (Figure 4). From day 5 of culture, the morphology of d-hMSCs under osteogenic condition and d-hMSCs cultured on SLA surface could not be discerned as SEM images revealed the progressive deposition of the matrix, characterized by the appearance of circular nodule formation and decrease in cell number. By day 7 of cell culture, the basal surface of the titanium discs was covered by the cell population and evidence of mineralized crystalline-like structure became more evident. This appearance was clearly distinguishable from that of the cells cultured on the machined surface without osteogenic induction media.

### 3.2. Expression of Osteogenic Genes of d-hMSCs (RT-PCR)

**Figure 5:**
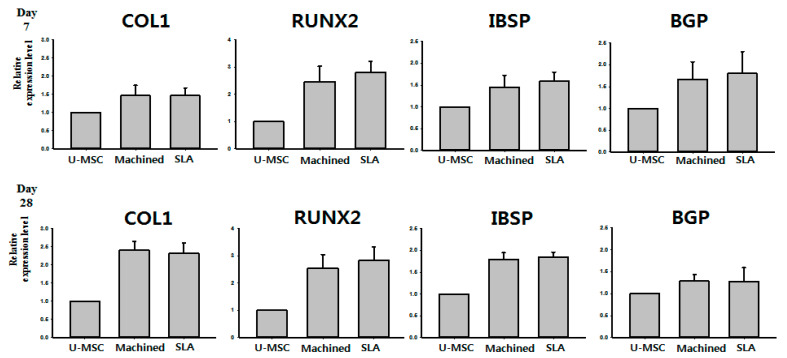
Molecular quantification of osteogenic differentiation of d-hMSCs on either cell plate (undifferentiated MSC, U-MSC), machined (machined) or SLA-treated surface (SLA) titanium discs.

RT-PCR analysis measured the expression of osteogenic genes including RUNX2, COL1, BGP and IBSP of d-hMSCs’ proliferation on differently surface-treated titanium discs after 7 days and 28 days of initial cell culture. D-hMSCs cultured on traditional cell plate were used as a control to normalize the results obtained in the RT-PCR analysis. From the results of day 7, it was clearly noted that d-hMSCs cultured on SLA-treated discs displayed greater bone-related gene expression than d-hMSCs cultured on conventional cell plate and machined titanium discs, although there was no statistically significant difference among the groups (*p* > 0.05). On the other hand, COL1, a representative early osteogenic marker, was rather lesser evident on SLA-treated surfaces than machined surfaces, suggesting progression of osteogenic differentiation. Although statistically not important, 28 days after initial cell culture, IBSP and BGP expression of d-hMSC culture on SLA-treated titanium discs was also observed to be greater than that on machined titanium discs (*p* > 0.05) (Figure 5).

### 3.3. Radiological Analysis 

**Figure 6:**
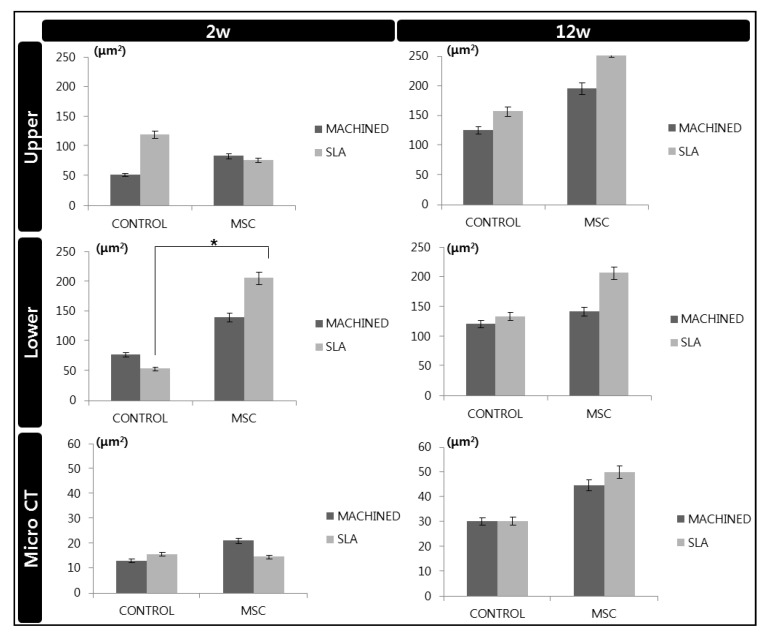
Newly formed bone (upper and lower) measured from X-ray images and within ROI from micro CT images. *: *p* < 0.05.

As aforementioned, new bone volume (μm^2^) was determined at two points from plain X-ray images, (a) upper and (b) lower. At 2 weeks, the new bone volume (upper) measured in the control group of SLA-treated surface discs was rather greater than that of the d-hMSCs group, showing the negative effect of d-hMSCs on new bone formation. However, in the 12 week group, such a negative effect had been compensated for, showing that the d-hMSCs group demonstrated greater new bone volume than the control group regardless of the surface type (*p* > 0.05). On the other hand, the new bone volume measured in the lower part of the implant (lower), the area in which the cells are most prone to be active due to the orientation of injection, displayed greater new bone formation in d-hMSCs group at both 2 weeks and 12 weeks. In particular, when comparing new bone volume (lower) measured from the SLA-treated surface of the control group and the d-hMSCs group, the d-hMSC group showed statistically significantly greater new bone volume than the control group (*p* < 0.05). Micro CT results which measured new bone volume within the ROI region are in accordance with the results of the measurement from plain X-ray images. Although statistically not significant, the d-hMSCs group at 12 weeks demonstrated a greater new bone volume than the control group regardless of the surface type (Figure 6).

### 3.4. Histological and Histomorphometric Analysis (H&E, Russell-Movat Pentachrome: BIC, BV)

**Figure 7:**
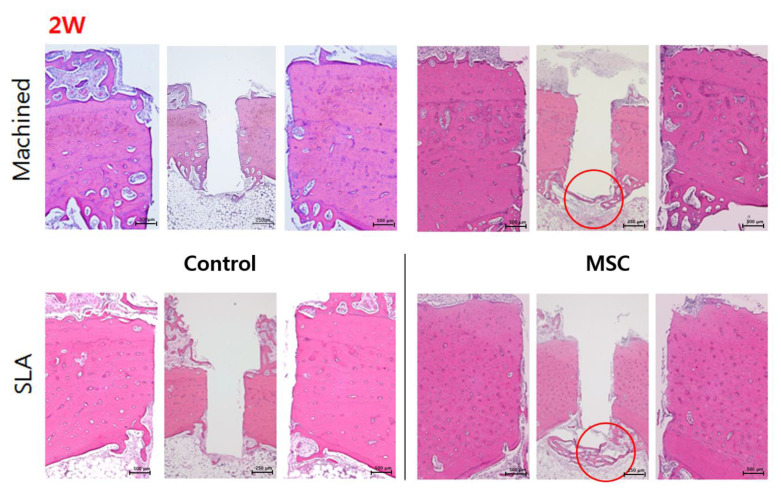

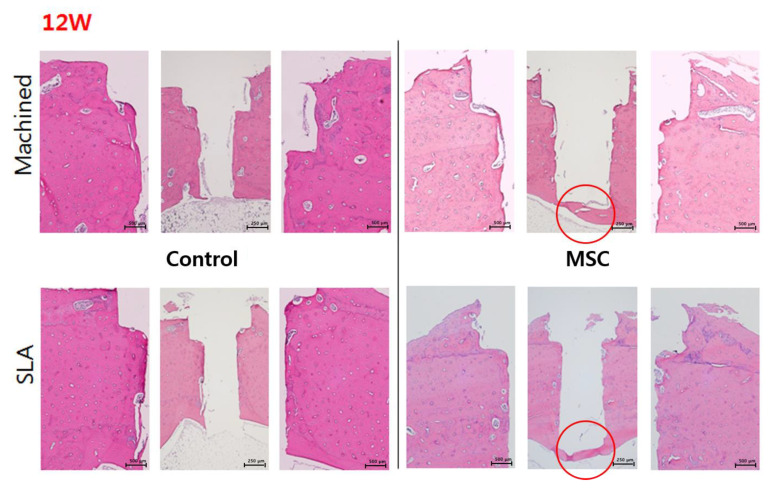
Light-microscopy views of the control group (without d-hMSCs) and the d-hMSCs group sacrificed at 2 and 12 weeks post-surgery (hematoxylin and eosin (H&E) stain, original magnification ×50, ×100; red circle indicates defect margin.

**Figure 8:**
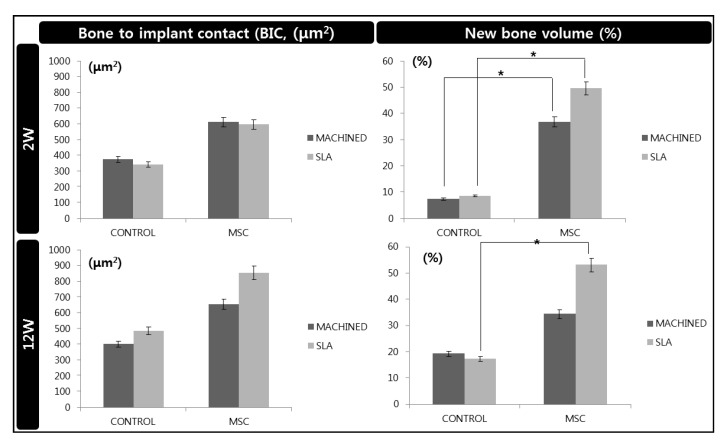
Histomorphometric analysis light-microscopy views of the control group (without d-hMSCs) and the d-hMSCs group sacrificed at 2 and 12 weeks post-surgery. *: *p* < 0.05.

None of the animals used in this study exhibited excessive inflammation, maintaining a healthy condition. Therefore, all samples were included for micro CT, histologic and histomorphometric analysis. At 2 weeks, regardless of the surface type of implant, a new bone interface connecting the surgically created defect was observed in the d-hMSCs group as a form of elongated bone cluster mixed with fibrocartilaginous callus-like tissue, whereas the clear defect margin was maintained in the control group, showing no sign of new bone formation along the defect margin. In the control group, a large amount of bubble-shaped adipose tissue consisting of many small droplets was identified below the defect margin and within the bone marrow. However, in the d-hMSCs group, this area filled seemed to be replaced by an extensive woven bone network, bridging from one side to the other side. There was no sign of immune response which may have resulted from heterogeneous human cell injection (Figure 7). At 12 weeks, more prominent continuous new bone formation was observed in the d-hMSCs group, showing complete ”bridging” of the defect. The bony bridge consisted of lamellar bone, characterized by consistent alignment of osteocytes. However, the thickness and quality of new bone did not seem to be different according to surface type. Conversely, the defect margin without d-hMSCs engrafting still showed opening of the defect, although a very small amount of new bone formation was observed near the defect margin. Additionally, the amount of adipose tissue which was abundantly found in the 2-week group was dramatically reduced in all groups and dense connective tissue was no longer identified (Figure 7). The results of the histomorphometric analysis are summarized in Figure 8. The average BIC values measured within the ROI for the control group (machined, SLA) were 375.25 (μm^2^) and 343.57(μm^2^) at 2 weeks, and 402.78 (μm^2^) and 486.34 (μm^2^) at 12 weeks, respectively. The average BIC values measured within the ROI for the d-hMSCs group (machined, SLA) were 613.03 (μm^2^) and 597.03 (μm^2^) at 2 weeks, and 655.67 (μm^2^) and 855.69 (μm^2^) at 12 weeks, respectively. There was no statistically significant difference in BIC values at any time point between the control group and the d-hMSCs group (*p* > 0.05).

As can be expected from the results of histologic analysis, new bone volume (BV%) measured within the ROI in the d-hMSCs group showed a significantly increased formation compared to that of the control group. The average BV (%) values measured within the ROI for the control group (machined, SLA) were 7.47% and 8.72% at 2 weeks, and 19.27% and 17.36% at 12 weeks, respectively. The average BV (%) values measured within the ROI for the d-hMSCs group (machined, SLA) were 36.90 (%) and 49.64 (%) at 2 weeks, and 34.39 (%) and 53.07 (%) at 12 weeks, respectively. At 2 weeks, regardless of surface type, the d-hMSCs group showed statistically significantly increased new bone formation compared to that of the control group (*p* < 0.05). Although the average BV (%) values did not differ significantly between the control group and the d-hMSCs group with machined surface at 12 weeks, there was significant difference in BV (%) values between the control group and the d-hMSCs group with SLA-treated surface.

**Figure 9:**
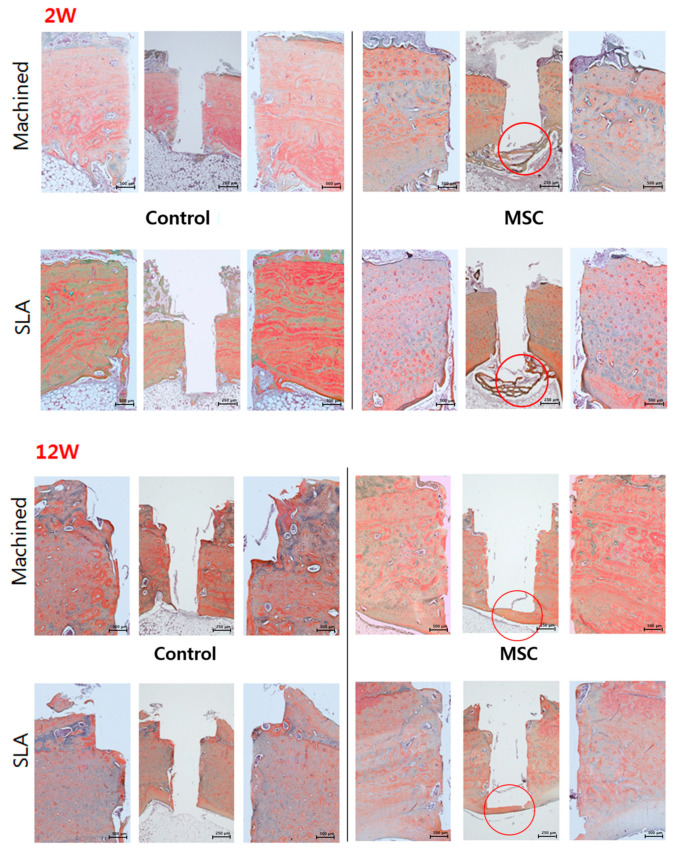
Light-microscopy views of the control group (without d-hMSCs) and the d-hMSCs group sacrificed at 2 and 12 weeks post-surgery (Russell–Movat pentachrome stain; original magnification ×50, ×100).

When comparing the maturity of bone depending on the time point, Russell–Movat **p**entachrome staining further confirmed that the d-hMSCs group at 12 weeks showed higher histological maturity of bone than the d-hMSCs group at 2 weeks. As shown in Figure 9, new bone network found in the d-hMSCs group at 2 weeks stained as green/yellow, indicative of woven bone, while new bone bridge found in the d-hMSCs group at 12 weeks stained as red, indicative of lamellar bone, and this color is similar to that of the intact bony tissue next to the defect (Figure 9).

### 3.5. Immunohistochemistry 

**Figure 10:**
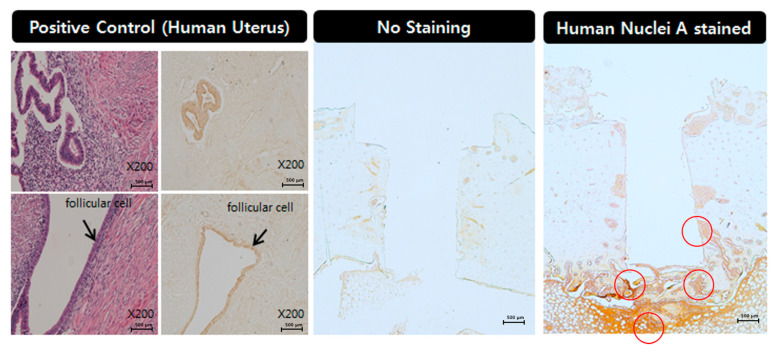

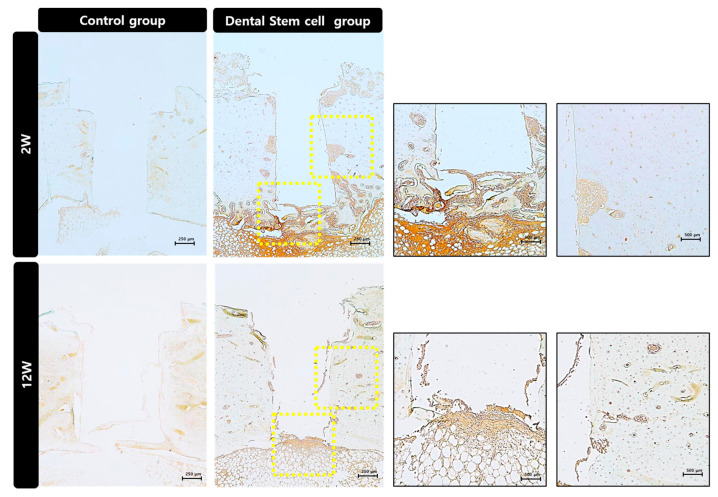
Immunohistochemical analysis of human nuclei A in the control group (without d-hMSCs) and the d-hMSCs group sacrificed at 2 and 12 weeks post-surgery (original magnification ×50, ×100).

The immunohistochemical analysis revealed that human nuclei A exhibited to moderate to strong expression in the d-hMSCs group at 2 weeks and the expression was mostly confined to the area below the implant, in which the d-hMSCs were most likely deposited. Partial expression was also identified alongside the body of implant. However, the level of expression was not as strong as that found in the bottom of the implant. At 12 weeks, human nuclei A still exhibited weak to moderate expression in the d-hMSCs group, showing the survival of the d-hMSCs injected, but the level of expression was weaker than that found at 2 weeks (Figure 10).

## 4. Discussion

Many contemporary studies have demonstrated therapeutic benefits of engrafted hMSCs for local bone regeneration without any significant side effects [20,21,22,23]. However, several studies have also reported that most engrafted hMSCs were not found in the target area and mostly died or were entrapped in the lung if intravenously injected, limiting the use of hMSCs as a routine clinical application [24]. Indeed, more preclinical research is required to accurately assess the therapeutic potential of hMSCs by mimicking site-specific clinical conditions. In this study, we attempted to investigate the behavior of d-hMSCs, which have more advantages than hMSCs obtained from bone marrow of the iliac crest, [25,26] in response to differently surface-treated implants, and also the effect of d-hMSCs on local osteogenesis around an implant depending on the different surface type, evaluated using a tibia model in rabbits.

The results from SEM analysis clearly showed that a more active d-hMSCs cellular response was identified on SLA-treated surfaces than machined surfaces and this difference became more discernible from day 5 of initial culture, showing circular nodule formation in a dispersed form. From day 7, mineralized crystalline-like structure was identifiable on SLA-treated surfaces and on machined surfaces with osteogenic induction media, while this feature could not be found with the cells cultured on machined surfaces without osteogenic induction media. These time-dependent, surface-dependent cellular morphological changes were confirmed quantitatively throughout the results of RT-PCR, showing greater bone-related gene expression including COL-1, RUX2, IBSP and BGP on SLA-treated surfaces although no statistically significant difference in the level of expression was identified between SLA-treated surfaces and machined surfaces (*p* > 0.05). These results were in accordance with those of our previous study, which reported the increased early osteogenic markers (COL1, RUNX2) with dental stem cell-derived induced pluripotent stem cells cultured on the SLA-treated titanium discs in comparison to machined titanium discs [27]. As reported in many previous studies [28,29,30], it has been generally accepted that the rougher the implant surface, the more active the hMSC cell response present in terms of adhesion to the surface and differentiation into osteoblast through activation of RhoA/ROCK pathway, which ultimately influences the transcription factor RUNX2 to control osteoblast differentiation and matrix mineralization [31,32]. Therefore, it was once more confirmed throughout this study that the cell response of d-hMSCs can be affected by the topography of implant materials and it may be further anticipated that manipulating topography of implant surfaces could control the fate of d-hMSCs for bone tissue engineering applications.

In addition to the in vitro analysis of d-hMSCs, in vivo cell transplantation studies were also performed, since the ultimate purpose of the present study was to evaluate if the d-hMSCs can survive on the defects and directly regenerate bone around an implant. Although calvarial defect models is a most frequently used defect model to assess in vivo osteogenesis, this model only evaluates intramembranous bone formation in a non-load bearing area [33]. Instead, we used a tibia defect model in the present study to accurately access the bone-forming ability around implants placed in load-bearing bone, which better mimics an actual clinical situation. Additionally, since it is well known that the choice of an appropriate scaffold, which should enable cell delivery, survival and attachment, is a pivotal factor in determining the success of cell transplantation, we used cytocompatible Matrigel^TM^, which does not directly contribute to bone formation, so that the pure osteogenic effect of cell transplantation could be deduced from the results of the d-hMSCs group.

According to the results of radiographic and histomophometric analysis, the osteogenic effect d-hMSCs around an implant was clearly identified, showing the greater new bone formation especially in the early phase of bone regeneration. In addition to direct conversion of hMSCs into osteoblast, Totelli et al. suggested ”indirect” action of hMSCs, insisting engrafted hMSCs may act as a “signaling center”, orchestrating the host response to the injury, especially in attracting host vasculature [34]. Sharing the same point of view, Le Blanc K et al. reported that such a ”trophic” or ”paracrine” effect of hMSCs can be critical in initial inflammatory phases as hMSCs are known to have anti-inflammatory functions [35,36]. In this sense, the significant increase in bone formation in the d-hMSCs group at 2 weeks can be interpreted in addition to their direct role as a source of new osteoblasts. These results are supported by the results of histomorphometric analysis, in which significant difference in new bone volume (%) was identified solely in the SLA-treated surface group at 12 week whereas a significantly increased osteogenic effect in the d-hMSC group was identified regardless of surface type. Again, it can be interpreted that the effect of d-hMSCs had been maximized in the early phase of bone regeneration, followed by additional osteogenesis effect of the SLA-treated surface at a later phase of bone regeneration.

Although it was not very hard to differentiate between newly formed bone quality of the d-hMSCs group at 2 weeks and that of the d-hMSCs group at 12 weeks from histologic images, the absolute value of new bone volume (%) did not show any difference. This may be due to spontaneous bone healing tendencies of a rabbit model as a result of endosteum and periosteal reaction. A. Piattelli et al. [37] reported that in 2 to 6 months, the newly formed bone around the implant was almost the same as the existing bone. In the present study, therefore, the results of the 12 week group might have been hindered by this limitation. To compare the maturity of bone, Russell–Movat pentachrome staining was employed depending on the time point, and verified that the d-hMSCs group at 12 weeks showed higher histological maturity of bone than the d-hMSCs group at 2 weeks.

Lastly, but very importantly, we assessed the survival time of engrafted d-hMSCs, since it was reported that most d-hMSCs are deemed to be dead or not function in target areas, which may result from various reasons including inappropriate cell density [38], regulation of microenvironment [39] and high oxygen tension [40]. The immunohistochemical analysis revealed that human nuclei A still exhibited moderate expression in the d-hMSCs group, showing the survival of the d-hMSCs up to 12 weeks post-engraftment, although the level of expression was weaker than that found at 2 weeks, which suggests the attenuating of the function of d-hMSCs at 12 weeks.

In summary, all parameters investigated in this study, including in vitro analysis, radiographic, histologic, histomorphometric and immunohistochemical analysis, were consistent with each other and revealed promising results that d-hMSCs enhance the new bone formation around an implant with the reinforced increase in osteogenic potential of SLA-treated surfaces. Nevertheless, it has to be admitted that translations of these results into clinical practice may encounter many challenges beyond safety concerns described previously [41]. Further study may be required in attempts to improve the longevity of the d-hMSCs and to determine the optimal conditions in which the osteogenic potency of d-hMSCs can be maximized.

## 5. Conclusions

Within the limitations of the present study, transplantation of d-hMSCs along with use of appropriate implant surface type can be used as promising tools to reconstruct an appropriate regenerative microenvironment, thereby increasing bone regeneration capacity. This method may further provide a possible strategy for significantly reducing time of current treatment modalities incorporated with dental implants.

## Figures and Tables

**Table 1:** RT-PCR primer information.

Gene Name	Gene ID	Sequences	Am Pliconlength (bp)
Hum: an GAPDH F	M33197.1	cgaccactttgteaagetea	203
Human GAPDH R		aggggagatteagtgtggtg	
Human IBSP F	NM_004967.3	egecaatgaatacgacaatg	196
Human IBSP R		gatgcaaagccagaatggat	
Human COL1A1 F	NM_000088.3	ggeccagaagaactggtaca	200
Human COLIA1 R		cgetgttettgcagtggtag	
Human BGLAP F	NM_199173.4	ggcagegaggtagtgaagag	194
Human BGLAP R		agcagagegacacectagac	
Human RUNX2 F	NM_001015051.3	agtgccagetgcatectatt	201
Hum an RUNX2 R		tgettgaattteccaagg	

**Table 2:** The experimental design and classification of animal groups.

No. of Samples	No. of Animals	2 Week Group		12 Week Group	
Control machined		10	5	10	5
Control SLA		10		10	
d-MSC machined		10	5	10	5
d-MSC SLA		10		10	

## Data Availability

The data presented in this study are available on request from the corresponding author. The data are not publicly available due to privacy or other restrictions.
